# Sodium–Glucose Cotransporter 2 Inhibitors in Valvular Heart Disease: Cardiovascular Benefit, Valve-Specific Effects, and Evidence Gaps—A Structured Narrative Review

**DOI:** 10.3390/jcm15166355

**Published:** 2026-08-17

**Authors:** Maria Rada, Maria-Laura Craciun, Ana-Maria Pah, Gheorghe Stoichescu Hogea, Daniela Gurgus, Milan Daniel Velimirovici, Dan Alexandru Surducan, Abdeldayem Mahmoud, Diana Utu, Cristiana-Adina Avram

**Affiliations:** 1 Department VI—Cardiology, Faculty of Medicine, “Victor Babeș” University of Medicine and Pharmacy of Timișoara, Eftimie Murgu Square 2, 300041 Timișoara, Romania; rada.maria@umft.ro (M.R.); laura.craciun@umft.ro (M.-L.C.); 2 Department XVI—Balneology, Medical Rehabilitation and Rheumatology, Faculty of Medicine, “Victor Babeș” University of Medicine and Pharmacy of Timișoara, Eftimie Murgu Square 2, 300041 Timișoara, Romania; gurgus.daniela@umft.ro; 3 Department I—Nursing, “Victor Babeș” University of Medicine and Pharmacy of Timișoara, Eftimie Murgu Square 2, 300041 Timișoara, Romania; milan.velimirovici@umft.ro; 4 Department III—Functional Sciences, Faculty of Medicine, “Victor Babeș” University of Medicine and Pharmacy of Timișoara, Eftimie Murgu Square 2, 300041 Timișoara, Romania; surducan.dan@umft.ro; 5 Department of Family Medicine, “Victor Babeș” University of Medicine and Pharmacy of Timișoara, Eftimie Murgu Square 2, 300041 Timișoara, Romania; mahmoodemadsalama@gmail.com; 6 Department II—Physiology and Pathophysiology, Faculty of Pharmacy, “Victor Babeș” University of Medicine and Pharmacy of Timișoara, Eftimie Murgu Square 2, 300041 Timișoara, Romania; diana.utu@umft.ro; 7 Department of Internal Medicine I, Faculty of Medicine, “Victor Babeș” University of Medicine and Pharmacy of Timișoara, Eftimie Murgu Square 2, 300041 Timișoara, Romania; avram.adina@umft.ro

**Keywords:** SGLT2 inhibitors, valvular heart disease, functional mitral regurgitation, tricuspid regurgitation, aortic stenosis, transcatheter aortic valve implantation, valve disease modification, cardiac remodeling

## Abstract

**Background/Objectives**: Native valvular heart disease (VHD) lacks established lesion-modifying pharmacotherapy. Sodium–glucose cotransporter 2 inhibitors (SGLT2is) improve heart-failure and cardiorenal outcomes, but their relevance to VHD differs by phenotype and treatment setting. **Methods**: MEDLINE/PubMed, Scopus, Web of Science Core Collection and Cochrane CENTRAL were searched from inception to 27 July 2026, together with trial registries, conference proceedings and reference lists. Evidence was synthesized by clinical setting, study design and mechanistic proximity to valve tissue. **Results**: Functional/secondary mitral regurgitation (MR) currently provides the most convincing valve-related signal: EFFORT and DEFORM showed concordant reductions in MR severity with favorable remodeling surrogates, consistent with HF-directed unloading rather than a primary leaflet effect. DapaTAVI provides the strongest hard-outcome evidence after the mechanical correction of aortic stenosis, reducing the 1-year death/worsening-HF composite in selected high-risk patients. In 28,940 HF patients from the SHEBAHEART registry, SGLT2i use was associated with reduced death/HF hospitalization and a 28% lower adjusted risk of tricuspid-regurgitation progression, but these data remain observational. Native aortic-stenosis progression is supported only by target-trial emulation, and no dedicated clinical evidence exists for native aortic regurgitation. **Conclusions**: SGLT2is should be integrated into guideline-directed HF therapy when indicated. In secondary MR, reassessment after optimized therapy should precede mitral intervention when clinically appropriate. A post-TAVI benefit should be interpreted as cardiorenal/HF protection, not valve modification. The current evidence does not justify SGLT2is solely to modify an untreated native valve lesion.

## 1. Introduction

Valvular heart disease occupies an unusual position in contemporary cardiology. In contrast to coronary artery disease and heart failure, no pharmacological therapy has been shown to reliably arrest or reverse the structural progression of a native valve lesion. Current management therefore relies on surveillance, the treatment of associated conditions and timely mechanical intervention. Statins, the most rigorously tested candidates for slowing calcific aortic stenosis, failed in three randomized trials, and no alternative agent has subsequently achieved an established lesion-modifying indication [1,2,3].

This therapeutic gap is increasingly consequential. Calcific aortic valve disease (CAVD) is the most common form of VHD in high-income countries, and its burden is increasing with population aging [4]. Aortic sclerosis—the earliest, hemodynamically silent stage—predicts cardiovascular morbidity and mortality even without significant obstruction [5]. Functional (secondary) mitral regurgitation (MR), meanwhile, is common in heart failure and is primarily a consequence of ventricular and atrial remodeling rather than intrinsic leaflet disease. It is therefore potentially responsive to optimized heart-failure therapy, although an improvement in MR severity should not be conflated with the direct modification of valve tissue [6].

The conceptual shift that makes pharmacological intervention plausible is the recognition that CAVD is an actively regulated disorder rather than passive degeneration. Valvular interstitial cells (VICs) can undergo osteogenic transdifferentiation under the influence of oxidized lipids, mechanical stress, innate immune activation and metabolic dysregulation, with the expression of RUNX2, BMP2, alkaline phosphatase and osteopontin [7]. NLRP3 inflammasome signaling and extracellular-matrix damage-associated molecular patterns contribute to this process in human VIC models [8,9]. These pathways create biological plausibility for anti-inflammatory and metabolic therapies, but plausibility alone does not establish a clinically relevant valve-specific effect.

Sodium–glucose cotransporter 2 inhibitors are prominent candidates in this context. Initially developed as glucose-lowering agents, they subsequently demonstrated cardiovascular and renal benefits that extend beyond glycemic control and are now integral to heart-failure therapy. The proposed mechanisms include natriuresis, altered myocardial substrate utilization, improved cellular energetics, reduced inflammation and oxidative stress, and reverse remodeling [10]. SGLT2is also attenuate vascular calcification through the suppression of endoplasmic-reticulum stress and TXNDC5-dependent osteogenic reprogramming [11]. More recently, canagliflozin attenuated calcification in an ex vivo human aortic-valve model and in experimental valve models [12]. This valve-proximal signal remains preclinical, and no in vivo human valve-lesion-modifying effect has been demonstrated.

Until recently, the clinical case rested largely on mechanistic convergence. Landmark SGLT2i outcome trials frequently excluded patients with severe valvular disease, leaving uncertainty in structural-heart populations. The evidence has since expanded. EFFORT and DEFORM tested SGLT2is in functional MR [13,14]; a target-trial emulation evaluated the progression of non-severe aortic stenosis [15]; and DapaTAVI provided the first large randomized clinical-outcome evidence after TAVI [16]. Additional observational studies have evaluated mitral TEER, native aortic stenosis, TAVI outcomes and bioprosthetic-valve failure [17,18,19,20,21], while recent meta-analyses have synthesized the post-TAVI evidence, but remain largely driven by one randomized trial and heterogeneous observational cohorts [22,23].

A useful clinical hierarchy emerges when valve-related effects are separated from patient-level outcomes. Functional/secondary MR currently provides the most convincing valve-related signal because two randomized trials show concordant reductions in regurgitant severity [13,14]. Post-TAVI treatment has the strongest hard-outcome evidence, but the stenotic valve has already been mechanically corrected [16]. Functional TR now occupies an intermediate, promising tier because a large longitudinal registry links SGLT2i use to better HF outcomes and less TR progression [24]. Native AS remains an observational disease-modification hypothesis [15,19], whereas native aortic regurgitation has no dedicated clinical evidence. This hierarchy is not a simple efficacy ranking: each phenotype answers a different question about HF therapy, post-interventional protection, or possible lesion modification. The review therefore synthesizes evidence according to the valve lesion, clinical setting, endpoint type and mechanistic proximity to valve tissue.

## 2. Materials and Methods

### 2.1. Design

This is a structured narrative review with a predefined search framework. A narrative design was selected because the available literature spans in vitro and animal studies, observational cohorts, target-trial emulations, small mechanistic randomized trials and one large outcome trial, with non-equivalent endpoints ranging from molecular signaling to echocardiographic surrogates and clinical events. A quantitative synthesis of all domains would therefore be methodologically inappropriate. PRISMA reporting and a de novo meta-analysis were not applied.

### 2.2. Information Sources and Search Strategy

MEDLINE/PubMed, Scopus, Web of Science Core Collection and the Cochrane Central Register of Controlled Trials were searched from database inception to 27 July 2026. ClinicalTrials.gov and the WHO International Clinical Trials Registry Platform were searched for ongoing or unpublished studies. The Proceedings of the American College of Cardiology, the American Heart Association, the European Society of Cardiology and the Transcatheter Cardiovascular Therapeutics meetings (2020–2026) were reviewed for late-breaking studies and abstracts not yet available as full publications.

The search combined three concept blocks with Boolean AND. Terms within each block were combined with OR and adapted to the syntax of each database:

Block 1 (intervention): “sodium–glucose transporter 2 inhibitors” [MeSH] OR SGLT2 inhibitor* OR gliflozin* OR dapagliflozin OR empagliflozin OR canagliflozin OR ertugliflozin OR sotagliflozin OR enavogliflozin.

Block 2 (condition): “heart valve diseases” [MeSH] OR valvular heart disease OR aortic stenosis OR aortic sclerosis OR calcific aortic valve disease OR mitral regurgitation OR tricuspid regurgitation OR rheumatic heart disease OR valve calcification OR valvular interstitial cell*.

Block 3 (setting, applied as an optional expansion): transcatheter aortic valve implantation OR TAVI OR TAVR OR transcatheter edge-to-edge repair OR TEER OR valve replacement OR bioprosthe*.

### 2.3. Eligibility Criteria

The eligible evidence comprised randomized controlled trials; target-trial emulations and propensity-matched cohorts; prospective or retrospective cohorts reporting valve, echocardiographic or clinical endpoints; mechanistic studies using human or animal VICs; animal models of valve disease; and systematic or narrative reviews used solely for contextualization and citation tracking. Only English-language reports were included.

Case reports, case series with fewer than 10 participants, studies in which valve disease was neither an eligibility criterion nor a reported endpoint, editorials without primary data and non-peer-reviewed preprints were excluded from the evidentiary synthesis. Conference abstracts were retained only when they supplied otherwise unavailable data and were explicitly identified as non-full-text evidence.

### 2.4. Study Selection and Synthesis

Records were screened by title and abstract, followed by a full-text assessment of potentially relevant reports. Backward citation tracking was performed from the included studies and relevant reviews, and forward citation tracking was performed for pivotal trials. The data were organized in a structured evidence matrix containing the design, population, valve lesion, intervention, follow-up, endpoint definition, numerical effect estimate and principal limitations. Key numerical estimates were checked against the full report or an official trial registry where available.

The evidence was synthesized by valve lesion and clinical setting. Within each domain, randomized evidence was considered first, followed by quasi-experimental designs, conventional observational cohorts and preclinical studies. Clinical endpoints were distinguished from imaging or biomarker surrogates, and the patient-level cardiovascular benefit was distinguished from evidence of direct valve-lesion modification.

### 2.5. Assessment of Certainty

Formal GRADE ratings were not assigned because this was not a systematic review. Instead, each domain was characterized by the strongest available design, the clinical or surrogate nature of the endpoint, the main threats to validity and the inference that the evidence could reasonably support. 

**Figure 1:**
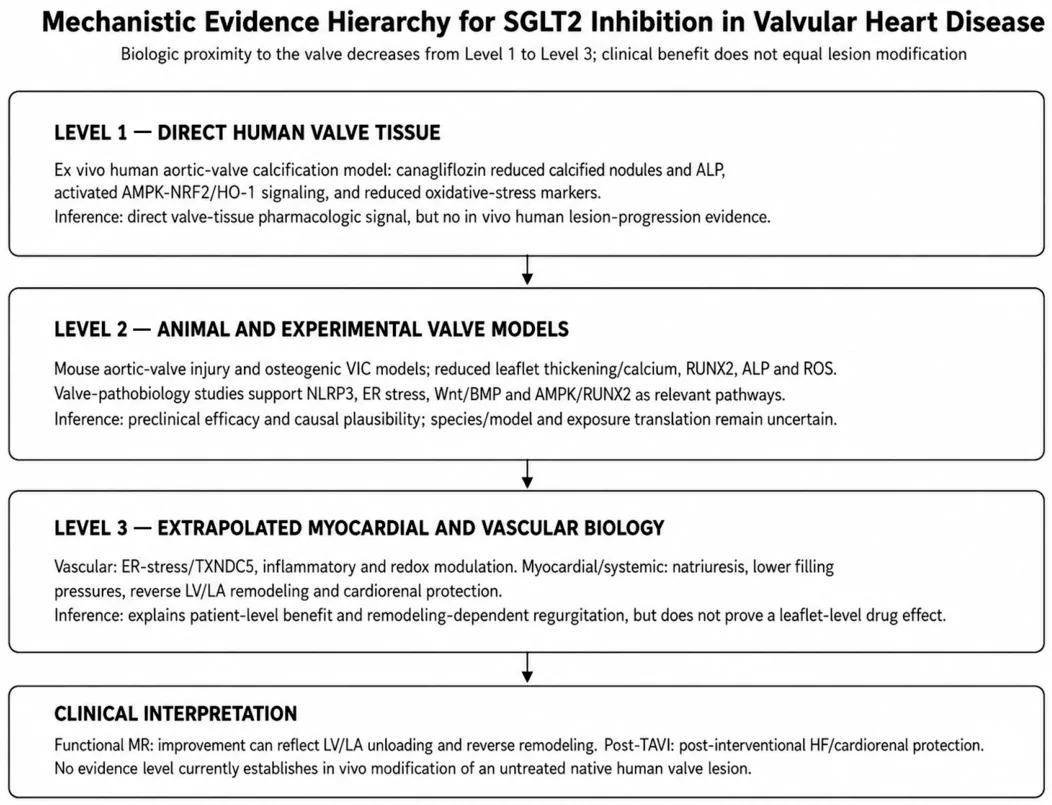
Mechanistic evidence hierarchy for SGLT2 inhibition in valvular heart disease. Level 1 comprises direct SGLT2i-specific ex vivo human valve evidence [12]; Level 2 comprises experimental valve and VIC models [7,8,12,25,26,27,28,29,30,31]; and Level 3 comprises mechanisms extrapolated from vascular and myocardial biology [10,11,32,33]. Functional MR improvement may result from ventricular/atrial unloading and reverse remodeling, whereas post-TAVI benefit is best interpreted as post-interventional cardiorenal and HF protection. None of these levels establishes in vivo modification of an untreated native human valve lesion. ALP, alkaline phosphatase; AMPK, AMP-activated protein kinase; ER, endoplasmic reticulum; HF, heart failure; LA, left atrial; LV, left ventricular; MR, mitral regurgitation; NRF2, nuclear factor erythroid 2-related factor 2; ROS, reactive oxygen species; RUNX2, runt-related transcription factor 2; TAVI, transcatheter aortic valve implantation; TXNDC5, thioredoxin domain-containing protein 5; VIC, valvular interstitial cell.

**Figure 2:**
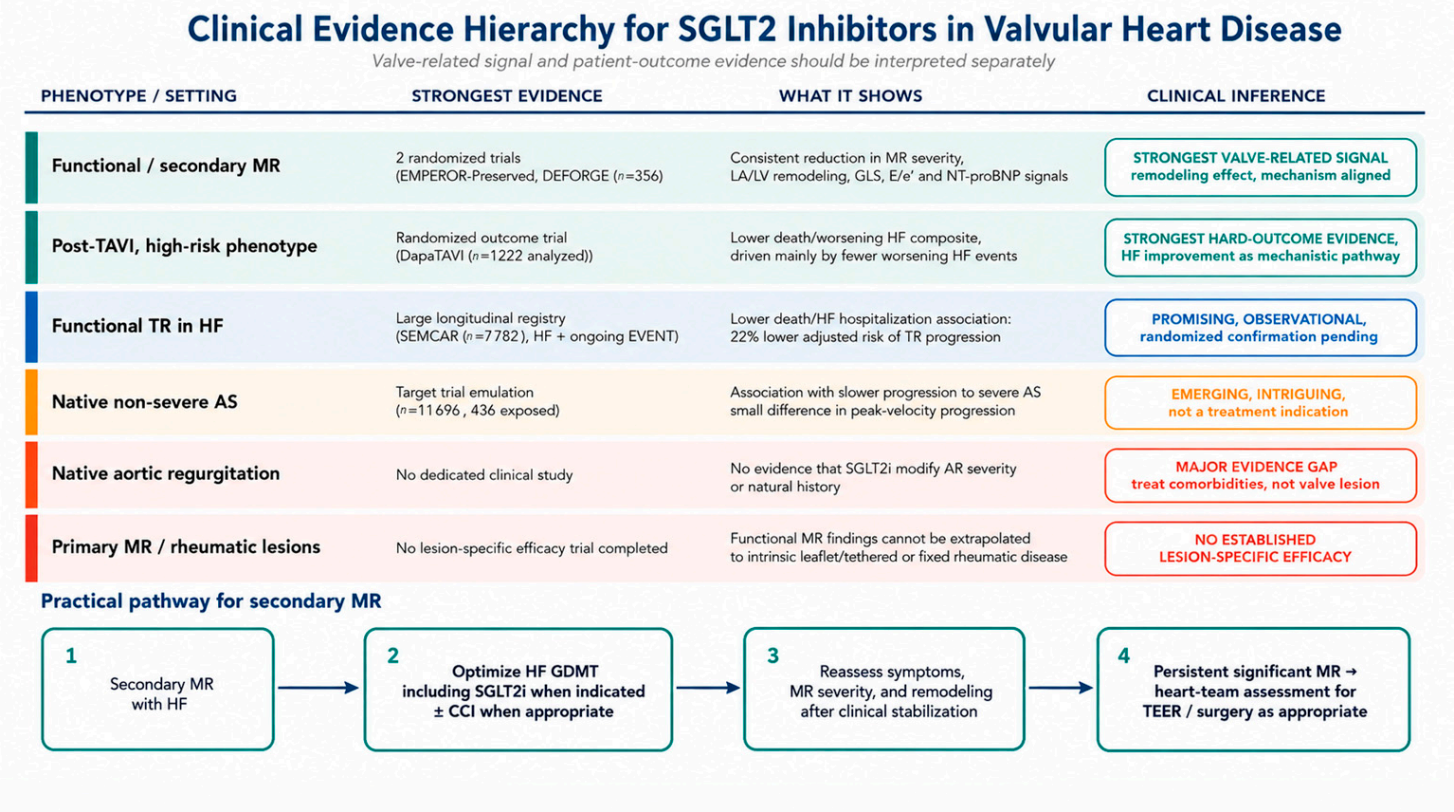
Clinical evidence hierarchy and practical pathway across valvular phenotypes. Functional/secondary MR provides the strongest valve-related randomized signal, whereas DapaTAVI provides the strongest hard-outcome evidence after the mechanical correction of aortic stenosis [13,14,16]. Large amounts of SHEBAHEART registry data place functional TR in a promising observational tier, with randomized confirmation awaited from EVENT [24,34]. Native AS remains an emerging, unconfirmed disease-modification hypothesis [15,19], while native aortic regurgitation lacks dedicated clinical evidence. The lower pathway summarizes the contemporary sequence for secondary MR: optimize HF GDMT (including SGLT2is when indicated and CRT when appropriate), reassess MR and remodeling, then consider TEER or surgery for persistent significant MR [35,36]. AS, aortic stenosis; GDMT, guideline-directed medical therapy; HF, heart failure; MR, mitral regurgitation; TAVI, transcatheter aortic valve implantation; TEER, transcatheter edge-to-edge repair; TR, tricuspid regurgitation.

Figure 1 and Figure 2 are original conceptual syntheses created by the authors from the reviewed literature. They contain no pooled patient-level data and are not reproductions or adaptations of published figures. Figure 1 organizes the mechanistic evidence by proximity to human valve tissue (direct ex vivo human-valve evidence, animal/experimental valve models, and extrapolated myocardial or vascular biology); Figure 2 maps the design strength to the inference supported.

## 3. Results

### 3.1. Overview of the Evidence Base

The evidence base is clinically heterogeneous, but can be ordered by phenotype. Functional/secondary MR has the most convincing valve-related randomized signal, whereas post-TAVI care has the strongest randomized hard-outcome evidence. Functional TR follows with a large observational SHEBAHEART signal and an ongoing dedicated randomized trial. Native AS progression remains hypothesis-generating, and native aortic regurgitation represents a major evidence gap. This ordering reflects the proximity and clinical meaning of the endpoint rather than a single universal certainty scale. Table 1 summarizes the principal clinical studies.

### 3.2. Mechanistic Substrate: An Evidence Hierarchy for Valve-Directed Plausibility

#### 3.2.1. Level 1: Direct Evidence in Human Valve Tissue

The most valve-proximal SGLT2i-specific evidence remains preclinical. In an ex vivo human aortic-valve calcification model, canagliflozin reduced calcified-nodule formation and alkaline-phosphatase expression, activated AMPK-NRF2/HO-1 signaling and reduced oxidative-stress markers [12]. This direct human-tissue signal is biologically important, but does not establish the in vivo target engagement, the durability, the hemodynamic benefit or the slowing of an untreated native valve lesion.

#### 3.2.2. Level 2: Animal and Experimental Valve Models

In experimental valve systems, canagliflozin reduced the aortic-valve velocity, leaflet thickening and calcium deposition in a mouse wire-injury model and attenuated RUNX2, alkaline phosphatase, calcium nodules and oxidative stress in osteogenic VICs [12]. Broader human and experimental valve studies implicate NLRP3, BMP/Wnt, ER stress, oxidative stress, metabolic reprogramming and the AMPK/RUNX2 axis in osteogenic transdifferentiation [7,8,25,26,27,28,29,30,31]. These data support causal pathways, but the species, injury model, exposure and timescale limit the translation to slowly progressive human CAVD.

#### 3.2.3. Level 3: Mechanisms Extrapolated from Myocardial and Vascular Biology

The largest mechanistic evidence base is indirect. Vascular models show ER-stress/TXNDC5 and inflammatory/redox modulation [11,32], while HF studies support natriuresis, lower filling pressures and reverse LV/LA remodeling [10,33]. These mechanisms are highly relevant to functional regurgitation and post-TAVI HF outcomes, but do not prove a leaflet-level action. Accordingly, myocardial and vascular biology should be used to explain plausibility and patient-level benefit, not to infer native-valve modification. The hierarchy of mechanistic evidence linking SGLT2 inhibition to valvular pathobiology, together with the principal inferences and limitations at each level, is summarized in Table 2.

### 3.3. Calcific Aortic Valve Disease

This is the domain of greatest theoretical interest and greatest evidentiary uncertainty. In a target-trial emulation of 11,698 patients with aortic sclerosis or non-severe aortic stenosis and at least 12 months of echocardiographic follow-up, SGLT2i initiation was associated with less progression to severe stenosis over five years (4.6% vs. 10.9%; adjusted HR: 0.61, 95% CI: 0.39–0.94) [15]. The annualized peak-velocity progression was also smaller (0.03 vs. 0.05 m/s/year; *p* = 0.0005), whereas differences in the aortic-valve area and dimensionless index did not reach conventional statistical significance [15].

The design merits both credit and scrutiny. Target-trial emulation specifies the eligibility, treatment assignment and follow-up to approximate a hypothetical randomized trial and reduces immortal-time and prevalent-user biases [37]. However, only 458 participants were exposed to SGLT2is, the confidence intervals were wide, the secondary echocardiographic measures had substantial missingness and residual confounding remains possible. Greater clinical contact among the treated patients may produce surveillance or healthy-adherer effects, whereas the higher prevalence of diabetes, heart failure and kidney disease among SGLT2i users could bias against benefit. A separate propensity-matched analysis in degenerative aortic stenosis reported a lower mortality and fewer valve replacements, but lacked systematic serial echocardiography and therefore did not directly measure hemodynamic progression [19].

The appropriate conclusion is deliberately narrow: native AS has generated a credible signal for slower progression, but its evidence is materially weaker than the randomized functional-MR and post-TAVI domains and is now also less developed clinically than the large functional-TR registry evidence. The statin experience—strong biological rationale and supportive observational data followed by neutral randomized trials [1,2,3]—is a direct warning against equating plausibility or target-trial emulation with the disease-modifying efficacy. SGLT2is should therefore not be prescribed solely to slow native AS outside a clinical trial.

### 3.4. Functional Mitral Regurgitation

Functional/secondary MR currently provides the most convincing valve-related signal for SGLT2 inhibition. It should not be viewed as an experimental leaflet-directed therapy; rather, SGLT2is are an integral component of contemporary HF treatment when indicated. EFFORT randomized 128 patients with functional MR and HF with mildly to moderately reduced EF to ertugliflozin or a placebo. At 12 months, EROA decreased with ertugliflozin and increased with the placebo (−0.05 ± 0.06 vs. +0.03 ± 0.12 cm^2^; *p* < 0.001), accompanied by a lower regurgitant volume, improved LV global longitudinal strain, favorable LA remodeling and improvement in the NYHA class [13]. DEFORM randomized 104 patients with moderate or severe functional MR to dapagliflozin or a control for 12 weeks; EROA decreased more with dapagliflozin (−0.074 ± 0.099 vs. −0.030 cm^2^; *p* = 0.008), with favorable changes in the regurgitant volume, E/e′, LAVI and LVEF [14].

The concordant changes in the MR severity, filling-pressure surrogates, atrial remodeling and ventricular function strongly support an unloading/reverse-remodeling mechanism rather than a primary pharmacological effect on mitral leaflets. Secondary MR is largely a manifestation of ventricular and/or atrial disease: the LV geometry, filling pressure, LA size, annular dimensions and leaflet tethering can all change with HF treatment. This interpretation is aligned with the 2025 ESC/EACTS VHD guideline and contemporary TEER frameworks, which place the optimization of HF therapy before definitive intervention whenever clinically feasible [35,36]. Observational data after SGLT2i initiation and after mitral TEER are directionally supportive, but remain non-causal [17,18].

The practical pathway is therefore explicit: first, confirm secondary MR in the context of HF; optimize guideline-directed HF therapy, including SGLT2is when indicated and cardiac resynchronization therapy when appropriate; then, reassess the symptoms, congestion, ventricular/atrial remodeling and quantitative MR after clinical stabilization. Patients with persistent significant symptomatic MR, despite optimized therapy, should proceed to heart-team evaluation for TEER or surgery according to the anatomy, ventricular status and guideline criteria [35,36]. SGLT2is are not an independent treatment for MR, and their initiation should never delay timely intervention in a patient who already meets procedural criteria.

### 3.5. Tricuspid Regurgitation

Early evidence for TR was limited to a small observational cohort in which moderate to severe TR did not improve over 6 months after SGLT2i initiation [18]. The evidence base has now changed substantially. In the SHEBAHEART big-data registry, 28,940 patients with HF contributed 79,313 echocardiograms over longitudinal follow-up. SGLT2i use was associated with a lower risk of death or HF hospitalization (adjusted HR: 0.79, 95% CI: 0.69–0.92), with consistent associations across TR strata [24]. Among 14,679 patients with serial echocardiography, SGLT2i use was associated with a 28% lower adjusted risk of TR progression (HR: 0.72, 95% CI: 0.58–0.90) and a 36% lower adjusted risk of worsening pulmonary pressures (HR: 0.64, 95% CI: 0.53–0.77) [24].

These findings elevate functional TR into a promising intermediate evidence tier, above native AS in current clinical relevance, but they remain observational and cannot establish causality or a direct tricuspid-valve effect. Residual confounding, treatment-selection effects and incomplete quantitative right-heart measurements remain important limitations; the findings are most applicable to secondary/functional TR in HF [24,38]. The ongoing randomized Enavogliflozin Outcome Trial in Functional Tricuspid Regurgitation (EVENT; NCT06027307) is testing enavogliflozin against a placebo and should provide prospective clinical and echocardiographic confirmation [34]. Until randomized data are available, SGLT2is should be used as HF therapy when indicated rather than prescribed as an independent TR-specific treatment.

### 3.6. Primary Mitral Regurgitation and Aortic Regurgitation

Native aortic regurgitation represents one of the clearest evidence gaps in this field. No dedicated randomized or observational study has evaluated whether SGLT2is alter AR severity, LV remodeling attributable specifically to AR, or the natural history of the native lesion. The same absence of lesion-specific evidence applies to primary degenerative MR. These lesions are driven by intrinsic leaflet, chordal, annular or aortic-root pathology and cannot be inferred to respond from trials of functional MR. SGLT2is may still be indicated for concomitant HF, CKD or diabetes, but their role in native AR is limited to the treatment of the patient’s comorbid HF/cardiorenal syndrome rather than valve-directed therapy.

### 3.7. Rheumatic Valve Disease

Rheumatic heart disease remains a major global cause of VHD, particularly in settings with limited access to surgery and transcatheter intervention. The proposed benefits of SGLT2is include hemodynamic unloading, myocardial and atrial remodeling, and the modulation of inflammatory or fibrotic pathways [39]. However, established commissural fusion and leaflet thickening are fixed structural lesions, and there is no mechanistic basis to expect SGLT2is to reverse advanced rheumatic mitral stenosis.

No completed clinical trial has evaluated SGLT2is in rheumatic valve disease. Dapa-Rhemis (NCT05618223) is evaluating dapagliflozin in rheumatic mitral stenosis, and NCT06097585 is evaluating gliflozins in heart failure associated with regurgitant rheumatic disease [39]. These studies focus on hemodynamic, functional and biomarker outcomes. Until the results are available, the use of SGLT2is in rheumatic VHD should follow conventional heart-failure, diabetes or kidney-disease indications rather than a valve-specific rationale.

### 3.8. The Post-Interventional Setting: DapaTAVI and Beyond

The strongest evidence in this field concerns the outcomes after the mechanical correction of aortic stenosis rather than pharmacological treatment of the stenotic valve. TAVI relieves valvular obstruction, but residual myocardial, renal and systemic vulnerability can persist and contribute to heart-failure events after the procedure [40].

DapaTAVI was a pragmatic, randomized, open-label, blinded-endpoint trial conducted at 39 Spanish centers [16,40]. Eligibility required a history of heart failure plus at least one of the following: LVEF ≤40%, diabetes mellitus or eGFR 25–75 mL/min/1.73 m^2^. Dapagliflozin 10 mg daily was initiated within 14 days after discharge. Of 1257 randomized patients, 1222 were included in the primary analysis. At 1 year, all-cause death or worsening heart failure occurred in 15.0% with dapagliflozin and 20.1% with standard care (HR: 0.72, 95% CI: 0.55–0.95; *p* = 0.02); worsening heart failure was reduced, whereas the all-cause mortality alone was not significantly different. Genital infection and hypotension were more frequent with dapagliflozin [16].

The clinically appropriate framing is therefore post-interventional heart-failure and cardiorenal protection in a selected high-risk TAVI population. Patients who most closely resemble DapaTAVI are older TAVI recipients with previous heart failure and concomitant LV systolic dysfunction, diabetes or moderate renal impairment. In such patients, dapagliflozin can be considered in the context of their overall HF/cardiorenal indication and safety profile. TAVI itself, however, should not be treated as a stand-alone indication for routine SGLT2i initiation in an otherwise unselected recipient, because that population was not tested in DapaTAVI.

This distinction also prevents a mechanistic overinterpretation: the obstructive native valve lesion had already been mechanically treated before the randomized comparison. The observed benefit is therefore most consistent with systemic, myocardial and renal effects after TAVI, not the modification of native aortic-valve biology. Decisions should incorporate the blood pressure, volume status, renal function, frailty, peri-procedural illness or fasting, and genital-infection risk.

Complementary observational data are supportive, but not causal. In 311 diabetic patients with severe aortic stenosis, a reduced ejection fraction and cardiac damage undergoing TAVI, SGLT2i use was associated with reduced major adverse cardiovascular events, mortality and heart-failure hospitalization over two years [20]. BIO-AS provided complementary myocardial expression/remodeling data in severe AS, but it did not test native-valve disease modification [41]. A propensity-matched study of 2297 TAVR recipients per group reported associations with a lower mortality (HR: 0.83, 95% CI: 0.71–0.97) and bioprosthetic-valve failure (HR: 0.62, 95% CI: 0.39–0.99) [21]. Recent meta-analyses generally report a lower mortality or heart-failure hospitalization after TAVI, but they pool DapaTAVI with heterogeneous observational studies and should not be interpreted as independent randomized confirmation [22,23]. EMPAVR (NCT06171802) is a double-blind, placebo-controlled trial evaluating empagliflozin after TAVI, with a change in the indexed left-ventricular mass by cardiac CT at six months as the primary endpoint [42]. Ongoing and planned trials evaluating SGLT2 inhibitors across valvular populations and addressing the principal remaining evidence gaps are summarized in Table 3.

### 3.9. Synthesis of Certainty Across Domains

The distribution of evidence across valvular domains is summarized in Figure 2 and Table 4.

## 4. Discussion

### 4.1. Clinical Interpretation by Patient Phenotype and Treatment Setting

From a valve-centered perspective, functional MR currently provides the most convincing pharmacologically modifiable signal. EFFORT and DEFORM are clinically important because they show concordant reductions in a dynamic, remodeling-dependent lesion during SGLT2i-based HF treatment [13,14]. The appropriate treatment target remains the ventricle and/or atrium, not the leaflet. Consequently, the actionable sequence is the optimization of GDMT and CRT when indicated, the reassessment of MR after stabilization, and heart-team evaluation for TEER or surgery only when significant symptomatic MR persists despite optimized therapy [35,36].

Post-TAVI evidence answers a different question. DapaTAVI provides the strongest randomized hard-outcome evidence in this review, but only after the stenotic valve has been mechanically corrected. Its benefit should therefore be framed as post-interventional HF/cardiorenal protection in patients resembling the enrolled phenotype—previous HF plus LV systolic dysfunction, diabetes or renal impairment—not as evidence of valve modification or a universal prescription strategy for all TAVI recipients [16]. This distinction is essential in older patients, in whom hypotension, the volume status, the renal function, frailty and the infection risk materially influence the benefit–risk balance.

Functional TR now occupies an intermediate tier between the randomized MR/post-TAVI domains and the hypothesis-generating native-AS literature. SHEBAHEART provides a large, internally consistent observational association with both clinical outcomes and TR progression [24], but the absence of randomization prevents causal interpretation. EVENT is therefore a key next step [34]. In contrast, native AS remains an observational disease-modification hypothesis, and native AR has no dedicated SGLT2i evidence. This gradient—secondary MR and post-TAVI at the top, followed by TR, native AS and then native AR—best reflects current clinical maturity while preserving the distinction between surrogate valve improvement and hard outcomes.

### 4.2. The Statin Precedent

The trajectory of statins in calcific aortic stenosis is the most relevant cautionary precedent. Lipid deposition and oxidation are prominent in early CAVD, and observational studies suggested a benefit, yet randomized trials were neutral [1,2,3]. One explanation is that treatment began after the disease had transitioned from a lipid-driven initiation phase to self-propagating mineralization and matrix remodeling.

The same temporal issue may apply to SGLT2is. Even a biologically active intervention could fail if initiated after calcification has become mechanically self-reinforcing. A definitive trial should therefore enroll aortic sclerosis or mild stenosis rather than advanced disease, use a reproducible structural endpoint such as serial CT calcium scoring and follow patients long enough to detect clinically meaningful progression.

### 4.3. Mechanistic Evidence: What Is Direct and What Is Extrapolated

Mechanistic evidence should be weighted by proximity to the human valve. Direct SGLT2i-specific evidence is limited to ex vivo human valve tissue [12]; experimental valve/VIC models provide a second tier [7,8,12,25,26,27,28,29,30,31]; and the broadest evidence is derived from vascular or myocardial biology [10,11,32,33]. These levels support biological plausibility and explain the remodeling-mediated functional-regurgitation benefit, but none establishes in vivo lesion modification. Mechanistic coherence should therefore motivate translational and randomized studies rather than lesion-directed prescribing.

### 4.4. Methodological Considerations in the Observational Evidence

Because the aortic-stenosis progression signal is observational, its methodology requires careful interpretation. Target-trial emulation reduces several avoidable biases by explicitly specifying the eligibility, treatment assignment and follow-up [37], but it cannot eliminate unmeasured confounding, exposure misclassification or differential surveillance.

The observed annual difference in peak-velocity progression was small (0.03 vs. 0.05 m/s/year) relative to the variability in serial echocardiography [15]. Only 458 patients were exposed, secondary echocardiographic measurements were incomplete, and residual confounding cannot be excluded. These limitations explain why native AS should carry less clinical weight than functional MR, post-TAVI evidence or the large TR registry signal, despite the statistical significance of the target-trial emulation.

### 4.5. Research Priorities

The decisive lesion-modification experiment is an adequately powered randomized trial in aortic sclerosis or mild aortic stenosis, before advanced calcification becomes mechanically self-sustaining. The serial CT aortic-valve calcium, standardized echocardiographic progression, adjudicated progression to severe stenosis and time to valve intervention should be prespecified over multi-year follow-up. A translational substudy should assess whether clinically achieved SGLT2i exposure engages the AMPK-NRF2/HO-1 or other candidate pathways in human valve tissue, thereby linking pharmacokinetics to a structural endpoint [12].

Further priorities are clinical-endpoint trials in functional MR embedded within contemporary GDMT, with standardized pre- and post-optimization imaging; the completion of EVENT and the rheumatic-valve studies; and the completion of EMPAVR to clarify post-TAVI remodeling under double-blind, placebo-controlled conditions [34,42]. Studies in broader or lower-risk TAVI populations would be required before any recommendation could extend beyond the DapaTAVI phenotype.

### 4.6. Limitations of This Review

This structured narrative review remains susceptible to selection bias and did not use duplicate systematic screening, formal risk-of-bias instruments or GRADE certainty ratings. The English-language restriction may have excluded relevant rheumatic-heart-disease literature, and one MR/TR echocardiographic cohort is available only as a conference abstract [18]. Although the new SHEBAHEART analysis substantially expands the TR evidence base, it remains observational and vulnerable to residual confounding [24]. Several observational studies and meta-analyses also share overlapping data sources, limiting independence. These limitations reinforce the need to privilege randomized evidence, distinguish surrogate from clinical endpoints and avoid interpreting remodeling-associated changes as proof of direct valve-tissue modification.

## 5. Conclusions

The clinical role of SGLT2is in valvular heart disease is phenotype-dependent and, on current evidence, rests on a heart-failure and cardiorenal benefit rather than on the direct modification of valve tissue. Two settings are already actionable. In functional/secondary MR, concordant reductions in the regurgitant severity across two randomized trials reflect HF-directed unloading and reverse remodeling rather than a leaflet effect; SGLT2is should accordingly be part of guideline-directed HF therapy when indicated, with MR reassessed after medical and device optimization and persistent significant symptomatic MR referred to the heart-team for TEER or surgery. After TAVI, the randomized DapaTAVI evidence supports SGLT2is for post-interventional HF and cardiorenal protection in selected high-risk patients, not as valve modification.

For the remaining phenotypes, the evidence does not yet support valve-directed treatment: the functional TR signal is observational and awaits the ongoing EVENT trial, native AS remains hypothesis-generating, and native aortic regurgitation has no dedicated clinical evidence. The key clinical message is therefore singular—use SGLT2is for their established heart-failure, chronic-kidney-disease and diabetes indications, including within the HF optimization of secondary MR, but not solely to modify an untreated native valve lesion.

## Figures and Tables

**Table 1:** Principal clinical studies of SGLT2 inhibitors in valvular heart disease.

Study (Year)	Design	Population	*n*	Agent	Follow-Up	Primary Endpoint	Principal Finding
DapaTAVI [16] (2025)	Randomized, open-label, blinded endpoint (PROBE); 39 centers, Spain	Severe AS undergoing TAVI, with prior HF plus LVEF ≤40%, diabetes, or eGFR 25–75	1257 randomized; 1222 analyzed	Dapagliflozin 10 mg	1 year	Death from any cause or worsening HF	Primary composite: 15.0% vs. 20.1%; HR: 0.72 (95% CI: 0.55–0.95). Mortality alone was not significant; genital infection and hypotension were more frequent.
Shah et al. [15] (2025)	Target-trial emulation, retrospective EHR	Aortic sclerosis or non-severe AS, ≥12 months echo follow-up	11,698 (458 exposed)	Any SGLT2i	Up to 5 years	Progression to severe AS	4.6% vs. 10.9%; adjusted HR: 0.61 (95% CI: 0.39–0.94); peak-velocity difference small; observational.
EFFORT [13] (2024)	Multicenter, double-blind RCT; 6 centers, Korea	Functional MR, NYHA II–III, EF 35–<50%, EROA >0.1 cm^2^	128	Ertugliflozin	12 months	Change in EROA at 12 months	EROA: −0.05 vs. +0.03 cm^2^ (*p* < 0.001); lower regurgitant volume; improved LV GLS, LA remodeling and NYHA class.
DEFORM [14] (2025)	Prospective randomized, parallel-control	Moderate or severe functional MR, LVEF <60%, EROA ≥0.2 cm^2^	104	Dapagliflozin 10 mg	12 weeks	Change in EROA	EROA: −0.074 vs. −0.030 cm^2^ (*p* = 0.008); improved E/e′, LAVI and LVEF; consistent with unloading and remodeling.
Thakkar et al. [17] (2024)	Propensity-matched cohort (TriNetX)	Patients undergoing mitral TEER	1289 per arm	Any SGLT2i	12 months	Death or HF hospitalization	HR: 0.694 (95% CI: 0.617–0.780); observational.
Observational echo cohort [18] (2025)	Prospective single-center observational	Chronic HF initiating SGLT2is	169	Any SGLT2i	6 months	Change in MR/TR grade	Moderate–severe MR fell 27% → 22% (*p* = 0.035); TR unchanged.
Loutati et al. [24] (2026), SHEBAHEART	Longitudinal big-data registry with time-dependent and sensitivity analyses	HF with and without significant functional TR	28,940; serial echo *n* = 14,679	Any SGLT2i	Median 3.5 years overall; 3 years for TR-progression analysis	Death or HF hospitalization; TR progression	Death/HF hospitalization aHR: 0.79 (95% CI: 0.69–0.92); TR progression aHR: 0.72 (95% CI: 0.58–0.90); observational.
Morel et al. [19] (2026)	Propensity-matched retrospective	Degenerative AS	10,912 per arm	Any SGLT2i	Median 1.22 years	All-cause death; SAVR/TAVR	Lower mortality and fewer SAVR/TAVR procedures; no systematic serial echo, so valve progression was not directly measured.
Morel et al. [21] (2025)	Propensity-matched retrospective cohort (TriNetX)	Patients after TAVR	2297 per arm	Any SGLT2i	Median 4.85 years	All-cause mortality; bioprosthetic-valve failure	Mortality HR: 0.83 (95% CI: 0.71–0.97); valve-failure HR: 0.62 (95% CI: 0.39–0.99); observational and dependent on coded outcomes.

**Table 2:** Evidence hierarchy for mechanisms linking SGLT2 inhibition to valvular pathobiology.

Evidence Level	Valve-Specific Evidence/Source	SGLT2i-Specific Findings	Inference and Key Limitation
Level 1: direct human valve tissue	Ex vivo human aortic-valve calcification model [12]	Canagliflozin reduced calcified nodules/ALP and activated AMPK-NRF2/HO-1.	Direct valve-tissue signal, but ex vivo only; no in vivo structural or hemodynamic endpoint.
Level 2: experimental valve models	Mouse aortic-valve injury and osteogenic VIC models [12]	Reduced valve velocity, leaflet thickening, calcium, RUNX2 and oxidative stress.	SGLT2i-specific efficacy in experimental valve models; translation to chronic human CAVD is uncertain.
Level 2: valve-pathobiology substrate	Human/experimental VIC and valve studies [7,8,25,26,27,28,29,30,31]	NLRP3, BMP/Wnt, ER stress, redox and AMPK/RUNX2 regulate osteogenic transition.	Defines relevant valve pathways; most studies are not SGLT2i interventions.
Level 3: vascular extrapolation	Vascular/cardiovascular models [11,32]	ER-stress/TXNDC5 and inflammatory/redox modulation.	Biologically plausible, but indirect; vascular calcification is not native-valve modification.
Level 3: myocardial/systemic extrapolation	HF remodeling and cardiorenal evidence [10,33]	Natriuresis, lower filling pressures and reverse LV/LA remodeling.	Directly relevant to functional MR/post-TAVI outcomes; supports treatment of the patient/ventricle, not a leaflet effect.

**Table 3:** Ongoing and planned trials of SGLT2 inhibitors in valvular populations.

Trial/Identifier	Setting	Agent	Principal Endpoint Focus
Dapa-Rhemis (NCT05618223) [39]	Rheumatic mitral stenosis	Dapagliflozin	Hemodynamic, functional biomarker
NCT06097585 [39]	HF with regurgitant rheumatic valve disease	SGLT2i	Hemodynamic, functional biomarker
EVENT (NCT06027307) [34]	HF with functional tricuspid regurgitation	Enavogliflozin	18-month composite clinical/echocardiographic outcome, including worsening TR
EMPAVR (NCT06171802) [42]	Severe symptomatic AS undergoing TAVI	Empagliflozin	Change in indexed LV mass by cardiac CT at 6 months

**Table 4:** Evidence strength and clinical interpretation by phenotype and treatment setting.

Clinical Phenotype/Setting	Strongest Evidence	Main Limitation	Supported Inference	Practical Management Implication
Functional/secondary MR with HF	Two randomized trials [13,14] plus VHD guidance [35,36]	Small samples; surrogate endpoints; short DEFORM follow-up	Most convincing valve-related signal: MR can improve through unloading/reverse remodeling; clinical-outcome benefit unproven.	Use SGLT2is within HF GDMT when indicated; optimize GDMT/CRT, reassess MR, then consider TEER/surgery if significant symptomatic MR persists.
Post-TAVI, DapaTAVI-like high-risk phenotype	Randomized outcome trial [16]	Selected population; open-label allocation	Strongest hard-outcome evidence after intervention; benefit driven mainly by fewer HF events.	Consider in patients resembling DapaTAVI and/or with established HF/CKD/T2DM indications; do not extrapolate to all TAVI recipients or native-valve modification.
Functional TR in HF	Large longitudinal SHEBAHEART registry [24]; EVENT ongoing [34]	Observational allocation; residual confounding; incomplete quantitative right-heart measures	Associated with fewer death/HF-hospitalization events and lower TR-progression risk; causality unproven.	Use as evidence-based HF therapy when indicated; await randomized confirmation before a TR-specific claim.
Mitral TEER population	Propensity-matched cohort [17]	Residual confounding	Supportive association with fewer clinical events.	Continue HF therapy; TEER is considered for persistent significant MR after optimization and heart-team assessment.
Native non-severe AS	Target-trial emulation [15]	Only 458 exposed; missing serial measurements; small peak-velocity difference; residual confounding	Emerging, hypothesis-generating signal for slower progression.	Do not prescribe solely to slow AS outside a trial; randomized structural-endpoint confirmation is required.
Native aortic regurgitation	No dedicated clinical study	No lesion-specific data	Major evidence gap; no evidence that SGLT2is modify AR severity or natural history.	Treat concomitant HF/CKD/T2DM indications only; no valve-directed recommendation.
Primary degenerative MR	No dedicated lesion-modification study	Intrinsic leaflet/chordal pathology differs from secondary MR	Functional-MR evidence cannot be extrapolated to primary MR.	Follow guideline-based surveillance; intervene when indicated. Use SGLT2is only for independent HF/cardiorenal indications.
Rheumatic valve disease	No completed dedicated efficacy trial [39]	Clinical data pending	Hemodynamic/biological rationale only.	Use conventional HF/CKD/T2DM indications or clinical trials; no valve-specific recommendation.

## Data Availability

No new data were generated or analyzed in this study. All information was derived from previously published studies, which are cited within the manuscript.

## Abbreviations

The following abbreviations are used in this manuscript:

ALP	Alkaline phosphatase
AMPK	AMP-activated protein kinase
AS	Aortic stenosis
ATF4	Activating transcription factor 4
BMP2	Bone morphogenetic protein 2
CAVD	Calcific aortic valve disease
CHOP	C/EBP homologous protein
CI	Confidence interval
CKD	Chronic kidney disease
EF	Ejection fraction
eGFR	Estimated glomerular filtration rate
EHR	Electronic health record
ER	Endoplasmic reticulum
EROA	Effective regurgitant orifice area
GDMT	Guideline-directed medical therapy
GLS	Global longitudinal strain
HF	Heart failure
HR	Hazard ratio
LA	Left atrial
LAVI	Left atrial volume index
LV	Left ventricular
LVEF	Left ventricular ejection fraction
MACE	Major adverse cardiovascular events
MAPK	Mitogen-activated protein kinase
MMP-9	Matrix metalloproteinase 9
MR	Mitral regurgitation
NLRP3	NOD-, LRR- and pyrin domain-containing protein 3
NRF2	Nuclear factor erythroid 2-related factor 2
NYHA	New York Heart Association
ox-LDL	Oxidized low-density lipoprotein
PERK	Protein kinase R-like endoplasmic reticulum kinase
PROBE	Prospective randomized open blinded endpoint
RCT	Randomized controlled trial
ROCK1	Rho-associated coiled-coil-containing protein kinase 1
ROS	Reactive oxygen species
RUNX2	Runt-related transcription factor 2
SAVR	Surgical aortic valve replacement
SGLT2	Sodium–glucose cotransporter 2
SGLT2i	Sodium–glucose cotransporter 2 inhibitor
TAVI	Transcatheter aortic valve implantation
TAVR	Transcatheter aortic valve replacement
TEER	Transcatheter edge-to-edge repair
TGF-β	Transforming growth factor beta
TR	Tricuspid regurgitation
TXNDC5	Thioredoxin domain-containing protein 5
VHD	Valvular heart disease
VIC	Valvular interstitial cell
